# Antioxidant properties of nano-formulated carotenoids and natural phenolic compounds against oxidative stress-induced health diseases

**DOI:** 10.12688/openreseurope.21957.1

**Published:** 2026-01-06

**Authors:** Sotirios Kiokias

**Affiliations:** 1 Marie Skłodowska-Curie Actions, European Research Executive Agency, Brussels, Avenue Simon Bolivar 34, 1000, Belgium

**Keywords:** Nanomaterials, nanoencapsulation, carotenoids, tocopherols, polyphenols, oxidative stress.

## Abstract

Radical oxygen species formed in human tissue cells via many endogenous and exogenous pathways cause extensive oxidative damage which has been linked to various human diseases. This investigation focused on the antioxidant potential of nano-carriers loaded with dietary natural antioxidants as phytotherapeutic agents. Main aim of this scientific review is to update the most recent advances in the application of nanoencapsulation to natural antioxidants against aging-related oxidative stress and associated pathological conditions. It provides an overview of recent in vitro and vivo trials on the potential of several nanoencapsulated antioxidant compounds (such as carotenoids, tocopherols, flavonoids and phenolic acids) to retard oxidative changes linked to carcinogenesis, cardiovascular and other serious health damages.

## Plain language summary

Lipid peroxidation -through the formation of reactive oxygen species (ROS)- is a very harmful biological process that links to the development of many adverse effects for the human health (including cardiovascular diseases and carcinogenesis) Over the last decade, an increasing body of literature evidence has demonstrated the beneficial in vivo antioxidant properties of many phytochemicals (such as dietary carotenoids and phenolic compounds) against lipid peroxidation by trapping the chain reaction of free radicals in human organism.

However, the administration of natural antioxidants is very challenging due to instability, low bio-accessibility, and limited bioavailability. A recent body of literature has focused on the rapidly developed Nanoscience and Nanomedicine applications that offer efficient solutions against this technological and health related challenge.

This manuscript focuses on the antioxidant potential of various nano-carriers loaded with phytotherapeutic agents. The aim of this review is to update on the recent advances in the application of phytoantioxidant-based nano-delivery systems to combat the aging-related oxidative stress and associated pathological conditions.

## Introduction to nano-antioxidants

Lipid peroxidation, through the formation of reactive oxygen species (ROS), is a very harmful biological process that is linked to the development of many adverse effects on human health (including cardiovascular diseases and carcinogenesis) ( Kato *et al.*, 2023; Zheng *et al.*, 2023). Over the last few decades, an increasing body of literature has demonstrated the beneficial in vivo antioxidant properties of many phytochemicals (such as dietary carotenoids and phenolic compounds) against lipid peroxidation by trapping the chain reaction of free radicals in humans organism ( Aguirre-Joya *et al.*, 2020).

However, the administration of natural antioxidants is challenging due to their instability, low bio-accessibility, and limited bioavailability ( Gali *et al.*, 2023). The rapidly developing Nanoscience and Nanomedicine-based applications over the last decade have offered efficient solutions to this technological and health-related challenge. ( Moss *et al.*, 2018). More specifically, research on nano-engineered nanoparticles (NEPs) has revealed opportunities for the fabrication of nanocarriers that would efficiently deliver active nutrients in human tissues and cells ( Mishra *et al.*, 2022; Rosales *et al.*, 2024) and thereby protecting against neurodegenerative disorders ( Poovaiah *et al.*, 2018), cardiovascular diseases ( Taneja *et al.*, 2021), obesity, and type 2 diabetes ( Tsou *et al.*, 2019).

The term nano-antioxidants usually refers to natural compounds nanoformulated using recently developed material science and engineering methods ( Shakibaie *et al.*, 2024; Shetta *et al.*, 2024). Plant-based silver nanoparticles (AgNPs) synthesized from extracts of M. longifolia (Lamiaceae) and R. ellipticus (Rosaceae) are examples of such nano-based antioxidants ( Habib *et al.*, 2024). The surface area of nano- antioxidants plays an important role in their use, leading to improved radical scavenging and opportunities for nanoparticles functionalization. ( Eftekhari *et al.*, 2018; Jogaiah *et al.*, 2021). A body of literature has recently concluded that nano-encapsulation of natural antioxidants improves their stability and their application in different food products ( Ahmadian *et al.*, 2024; Luo *et al.*, 2025) while also enhancing their therapeutic properties as anti-inflammation, anti-cancer, anti-allergic, and anti-thrombotic agents ( Bhandari *et al.*, 2024; da Silva *et al.*, 2024).

Consequently, researchers have developed nutraceutical capsules containing various ranges of natural teprenoids and phenolic compounds, vita-mins, polyunsaturated fatty acids (PUFAs), and others ( Duhoranimana *et al.*, 2022). A few authors have suggested nanoliposomes as suitable encapsulants for entrapping and delivering various phytonutrients to target cells, thereby enhancing their bioaccessibility and bioavailability ( Liu *et al.*, 2024; Subramanian, 2021; Tu *et al.*, 2024). Bhat *et al.* (2024) provided an in-depth examination of the current state of polyphenol-loaded nano-carriers in breast cancer therapy, elucidating their potential as an innovative and precision-focused therapeutic modality for improving patient outcomes while mitigating side effects.

Several recent studies have explored the effect of nanoencapsulation on the bio-accessibility of polyphenols from botanical extracts ( Ferreira Dantas *et al.*, 2025; Kato *et al.*, 2023; Myint *et al.*, 2021).

**Figure 1.**
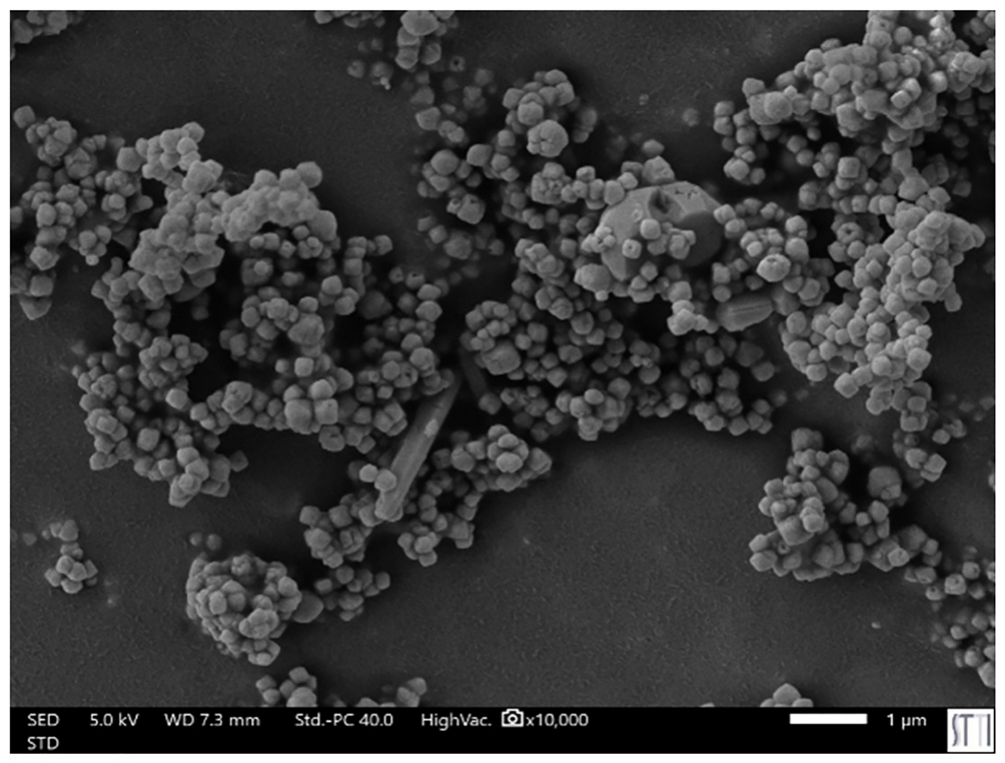
BaTiO3 nanomposites materials (Image taken by Scanning Electron Microscopy). *(kindly provided by Prof. J. Silvestre, University of Alicante/Spain)*.

The Figure 1 presents an image of the BaTiO3 nanocomposite materials (obtained by Scanning Electron Microscopy), which are used for various medical applications. In addition, Table 1 provides an overview of recently developed (<5 years) nano encapsulation/nano formulations as model systems for health-related application of natural antioxidants.

**Table 1.** Overview of recently developed (<5 years) nano encapsulation/nano formulations as model systems for health related application of natural antioxidants.

Natural antioxidant	Nano-formulated applications	Reference
**(1) Tocopherols**	i) Saponin-based nano-emulsions as α-toc delivery systems for dermal applications. ii) Solid lipid nanoparticles/NP-T3 for kidney/liver test iii) Squalene/α-toc-based nano-emulsions for induction of antigen-specific immune responses iv) Polycaprolactone-based nanocapsules containing moderate and high quantity of Toc.	Sheybani *et al.* (2023) Fu *et al.* (2023) Margaroni *et al.* (2024) Granata *et al.* (2025)
**(2) Carotenoids**	i) β-Carotene encapsulated in hydrogels to enhance the antioxidant activity ii) Zeaxanthin nanoencapsulated by use of .Cactus cladode mucilage (Opuntia monacantha) as a new natural structuring material iii) Nanoencapsulation of lycopene from Tomato Waste Using Chitosan and Alginate iv) Nanoencapsulation of Carotenoid-rich extract from cantaloupe melon (CE) in porcine gelatin (EPG) for exploring on hepatic retinol concentration	Darban *et al.* (2024) Boonlao *et al.* (2022) De Guzman *et al.* (2024) de Carvalho *et al.* (2023)
**(3) Flavonoids**	i) Synthesized polyvinylpyrrolidone (PVP) flavonoid. based nanoparticles ii) Silica nanoparticles (SiO2NPs) and natural zeolite nanoparticles (ZeNPs) as nano carriers to enhance the bioavailability of curcumin iii) Quercetin-loaded nano-emulsions based on olive oil iv) Encapsulating rutin in liposomes produced with hydroxypropyl methylcellulose (HPMC).	Hu *et al.* (2018) Moradi *et al.* (2023) Silva *et al.* (2023) Lopez-Polo *et al.* (2024)
**(4) Phenolic acids**	i) Nanoencapsulated Ferulic acid (FA) in suspensions (NC-FA) with ethylcellulose. ii) ρ-coumaric acid – Squid chitosan nanogel loaded with Syzygium aromaticum essential oil. iii) Rosmarinic acid (RosA) used to prepare green metallic-nanoparticles (Cu0.5Zn0.5FeO4 NPs) and encapsulated them using PEG polymer. iv) Rosmarinic acid loaded chitosan encapsulated graphene nanoparticles (RA- CH-G-NPOs) to enhance wound healing capacity	Rampelotto *et al.* (2022) Kamal *et al.* (2021) Imraish *et al.* (2024) Chhabra *et al.* (2020)

This study focused on the antioxidant potential of nanocarriers loaded with phytotherapeutic agents. The aim of this review is to update on the recent advances in the application of phytoantioxidant-based nano-delivery systems to combat age-related oxidative stress and associated pathological conditions. Therefore, the main focus of this analysis was to provide an overview of the most recent in vitro and in vivo studies exploring the therapeutic properties of nano-delivered carotenoids and various phenolic compounds. The next sections present the health protection potential of several encapsulated natural antioxidants, as reported by the most recent literature in this scientific field.

## Nano-tocopherols

Tocopherols are lipid-soluble phenolic antioxidants that are widely distributed in nature in a series of homologues (α-, β-, γ-, and δ-). They are known for their vitamin E content and well-established radical scavenging activities ( Kiokias *et al.*, 2018; Qiao *et al.*, 2019).

Several novel nano-encapsulation techniques applied in the food and pharmaceutical industries have been shown to provide higher bioavailability of vitamin E in humans. Nano-encapsulation of vitamin E has versatile advantages for site-specific targeted delivery and effective absorption in cells ( Kaur & Kaur, 2021). Recently, a few researchers ( Benbakrim *et al.*, 2025; Granata *et al.*, 2025) produced small solid nanodispersions of α- and δ-tocopherols and reported perfect encapsulation efficiencies and improved tocopherol stability in the tested systems. Similarly, Benbakrim *et al.* (2025) examined the possibility of preparing surfactant-free nanoencapsulation systems for tocopherols despite their low water solubility and poor surface activity.

Sheybani *et al.* (2023), however, did not observe higher antioxidant activity of nanostructured lipid carriers (NLCs) containing α-tocopherol compared to the control. Instead, their experiments concluded a higher synergistic antioxidant action when rosemary essential oil was added to α-tocopherol-based NLCs.

Recent studies have focused on nanoemulsions as delivery systems for tocopherols. Hong *et al.* (2024) (NEs) showed that multilayer nanoemulsions exerted statistically significant activities against oxidative deterioration and enhanced the in vitro bio-accessibility of α-tocopherol (ToC). Schreiner *et al.* (2023) reported similarly enhanced antioxidant effects when saponin-based nanoemulsions were developed as nanocarriers for α-tocopherol in dermal applications. In addition, Margaroni *et al.* (2024) produced a novel α-tocopherol-based self-emulsified nanoemulsion that can serve as a successful antigen delivery vehicle with potential for future vaccine-related applications.

Fu *et al.* (2023) explored the effects of nano-delivery systems (such as nanovesicles/NV-T3 and solid lipid nanoparticles/NP-T3) on the bioavailability and tissue biodistribution of vitamin E tocotrienols. Both tested nanodelivery systems showed elevated accumulation in the kidneys and liver (5-fold) compared to the control group, whereas selectivity for α-tocotrienol was evident for NP-T3. Overall, the study concluded that enhanced bioavailability and selective tissue accumulation of tocotrienol congeners when delivered via nanoencapsulation.

Olim *et al.* (2021) performed a very interesting biochemical study concluding that tocopherol-based cholesterol doxorubicin prodrug conjugates could offer promising nano formulations for use in breast cancer chemotherapy.

The potential of tocopherol-loaded nanoparticles to improve the water dispersibility of these hydrophobic compounds, along with their radical scavenging activity, has also been reported in vitro ( Hu *et al.*, 2018) and in vivo following oral administration in rats ( Simon *et al.*, 2016).

Sherif *et al.* (2024) conducted a clinical trial in Nile tilapia to examine the effect of dietary supplementation with vitamin C and tocopherol nanocomposites. The results showed improved values of antioxidant biomarkers in the fish body, confirming a clear protective effect against lipid peroxidation.

## Nano-carotenoids

Carotenoid pigments are a group of bioactive compounds that are of interest to food scientists, nutritionists, and food industries due to their anti-inflammatory and antioxidant effects ( Varzakas & Kiokias, 2016) Carotenoids are widespread in nature (in plants, algae, fungi, birds, etc.), and terpenoids are classified into two groups: the more hydrophobic carotenes (a,ß-carotenes, and lycopene) and the more polar xanthophylls (including lutein and zeaxanthin and others) ( Kiokias *et al.*, 2016).

However, carotenoids are susceptible to heat- or light-induced oxidative degradation ( Boonlao *et al.*, 2022) while also characterized by limitations in terms of water solubility, which makes their commercial applications quite challenging for food scientists and nutritionists. ( Permatasari *et al.*, 2024; Sereti *et al.*, 2025).

**Figure 2.**
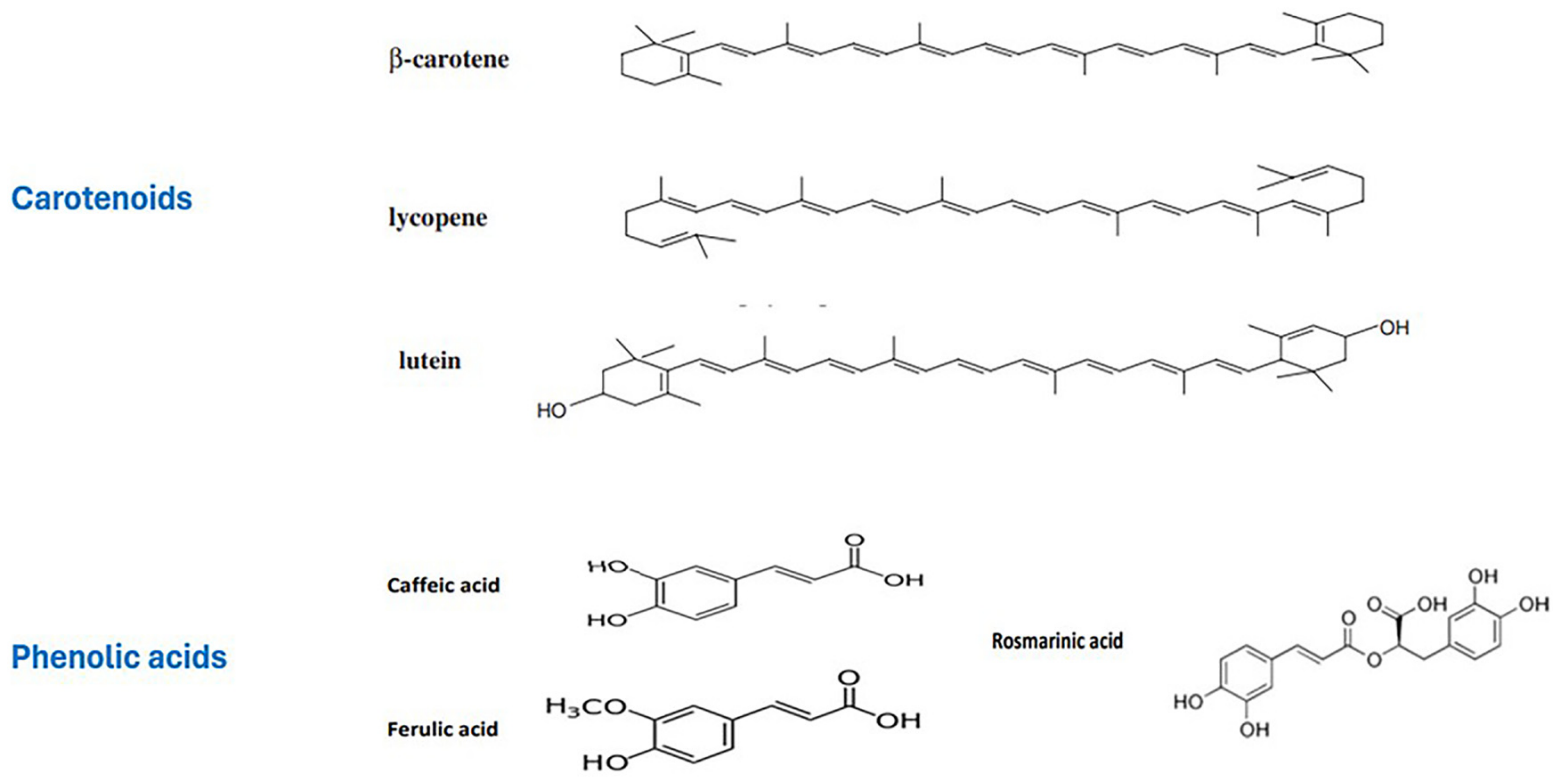
Chemical structures of several natural carotenoids and phenolic acids reviewed in the present study for their nano-encapsulation relevant data and health effects.

Nanocarotenoids have recently been prepared from both carotenoid extracts and standards by employing various preparation techniques to yield different nanostructures ( Sridhar *et al.*, 2021). In this regard, lipid-based nano-delivery cargos, such as nano-liposomal vehicles, surfactant-based nanocarriers, nanoemulsions (Nes), nano-structured lipid carriers (NLCs), and solid lipid nanoparticles (SLNs), as safe and attractive nanocarriers, are proving to be potent platforms for the protection of carotenoids against challenging conditions and offer efficient controlled release ( Rostamabadi *et al.*, 2019; Zare *et al.*, 2021). In this section, a range of carotenoids (beta-carotene, lycopene, lutein, and astaxanthin) were examined with reference to the developed nano formulations. The chemical structures of a few carotenoids examined in the framework of this review are given in Figure 2.

### Nano-carotenes and carotenoid extracts

β-carotene (βC) is the most well-known carotene with provitamin A activity, well-established antioxidant effects, and health-protective properties ( Awadalla *et al.*, 2025). In order to enhance the bioavailability and biological activities of β-carotene, a few researchers have recently investigated its nano-encapsulation in various models such as hydrogels ( Darban *et al.*, 2024) or protein polymer nanoparticles ( Ngew *et al.* (2025). Chaves *et al.* (2023) observed that β-carotene-loaded nanoparticles provide increased protection against oxidative damage in Drosophila melanogaster. de Oliveira *et al.* (2021) reported that nanoencapsulation of carotenoid-rich extract (CE) from cantaloupe melon (EPG) clearly enhanced the antioxidant potential of carotenoids with beneficial effects on human health.

Medeiros *et al.* (2019) encapsulated Cantaloupe melon carotenoids in porcine gelatin and demonstrated that such nanoencapsulation increased water solubility, thereby facilitating the technological application of these natural pigments in food-related products. De Carvalho *et al.* (2023) examined the effect of β-carotene nanoencapsulation in porcine gelatin (EPG) in Wistar rats and concluded that such a system can serve as a potential therapeutic factor in future novel strategies against various health diseases.

Pinna *et al.* (2025) focused on pumpkin extracted carotenoids and observed that carotenoid based nanocarriers led to ~90% encapsulation efficiency, demonstrating a great potential for future applications in novel food systems. In addition, Koo *et al.* (2023) reported a positive effect of nano-encapsulation on the bioavailability of the marine carotenoid fucoxanthin.

Lycopene is an -acyclic in structure-carotene that has been linked to beneficial antioxidant properties for the treatment of cardiovascular diseases and prostate cancer ( De Guzman *et al.*, 2024). A few researchers have recently shown that the nanoformulation of lycopene can efficiently enhance its bioavailability ( Deepika *et al.*, 2023). Recent studies have investigated various lycopene nanoencapsulation systems, such as (i) polymeric nanoparticles (NPs) for the controlled release of lycopene ( Misir & Kassama, 2025) and (ii) lycopene loaded nano micelles using the Caco-2 cell model ( Su *et al.*, 2025). Both studies confirmed that nanoencapsulation improves the solubility and bioavailability of lycopene, and that the developed nanoparticles could serve as efficient carriers for the delivery of this bioactive compound into intestinal epithelial cells.

### Nano-xanthophylls

Xanthophylls are yellow carotenoid pigments that occur widely in nature and are similar in structure to carotenes but contain oxygen atoms; therefore, they are less hydrophobic than -purely hydrocarbon-carotenes ( Kiokias *et al.*, 2008).

Bixin is a polar carotenoid that is quite sensitive to environmental conditions (e.g., heat-induced oxidative degradation and low water solubility) ( Enayati *et al.*, 2023). da Silva *et al.* (2024) reviewed the current literature on annatto-(Bixa orellana)based nanostructures for biomedical applications. Their analysis revealed that bixin-based nanostructures showed leishmanicidal, photoprotective, antioxidant, antimicrobial, and immunomodulatory efficacy and tissue regeneration potential with no or low toxic effects in the tested models.

Natural lutein is a yellow pigment that is mainly extracted from marigold flower preparations, and its consumption is related to a lower incidence of age-related macular degeneration (AMD) and cataracts ( Kiokias *et al.*, 2016). Sun *et al.* (2024) developed whey protein/chitosan-coated lutein nanoparticles, which were found to be highly efficient in improving the stability of this carotenoid, thereby enabling its technological and pharmaceutical applications. Similar nanoparticles were examined by Bhat *et al.* (2020), who concluded that whey proteins and chitosan polymers can ensure perfect encapsulation efficiency and enhanced inaccessibility of lutein in human cells.

Viana *et al.* (2023) administered lutein-loaded nanoparticles to female offspring rats and reported significant effects against oxidative stress and apoptosis, revealing the potential of nano encapsulated lutein as an alternative treatment for VPA-induced behavioral damage.

Zeaxanthin is a stereoisomer of lutein, which protects against oxidative damage and acts as a filter for ultraviolet light ( Kumari *et al.*, 2025). Horuz & Belibağlı (2019), following the incorporation of zeaxanthin nanoparticles and nanoemulsion (Zea-NE) in yogurt model systems, eventually reported enhanced carotenoid retention and bioavailability of nanoencapsulated lutein compared to the control samples. Similar conclusions were drawn by Boonlao *et al.*, 2022 concerning the protection that nanoencapsulation offers zeaxanthin from oxidative degradation, which offers new opportunities for the technological use of these carotenoids in food and nutraceutical products.

Astaxanthin (AXT) is a red-colored xanthophyll whose consumption has been reported to play a positive role in many human health problems owing to its antioxidant power ( Sun *et al.*, 2025). The positive effect of nanoscience on various health and technological applications of astaxanthin has been reviewed by Abdelazim *et al.* (2023). Pan *et al.* ( 2018a; 2018b) concluded that astaxanthin encapsulated in nanoliposomes (produced from 4% phospholipids) exhibited high antioxidant activity achieving a >80% encapsulation efficiency.

Other recent studies have confirmed the increased activity of nano encapsulated astaxanthin in various model systems, such as: (i) removal of harmful for human health heavy metals (i.e., cadmium, lead, etc.), as reported by Bharti *et al.* (2025), and (ii) higher DPPH free radical trapping properties and a stronger cytoprotective effect on oxidative cell damage. In addition, Jin *et al.* (2025) reported that astaxanthin-loaded polylactic acid-glycolic acid nanoparticles alleviated atherosclerosis in mice by suppressing macrophage ferroptosis induced by oxidized LDL via the NRF2/SLC7A11/GPX4 pathway (more info in Table 2).

**Table 2.** An overview of recent (<5 years) clinical animal trials on the health protective effects of various nano-antioxidant formulations.

Nano-Antioxidant Treatment	Main in vitro/in vivo effects	Reference
**(1)** Dietary supplementation of **Nile tilapia** with control, bulk vitamins and **nanocomposite** of **vitamins C and tocopherols**	The combined vtamin (C&E) treatment yielded Increased antioxidant and serum antibacterial (SAA) activities compared to bulk vitamins and control.	Sherif *et al.* (2024)
**(2)** Oral administration of **female Wistar rats** with **lutein-loaded nanoparticles** (5 mg/kg) or for 2 weeks	Nanolutein supplementation resulted into improved neurobehavior while also reduced oxidative damage and apoptosis bio-indicators	Viana *et al.* (2023)
**(3)** Astaxanthin-based **nanoparticles (NPs)** were synthesized and investigated for their effects against LDL oxidative degradation in mice	Treatment with lutein NPs presented higher in vitro and in vivo effects against oxidative damage and thereby better therapeutic effects than astaxanthin alone.	Jin *et al.* (2025)
**(4)** Control dietary supplementation of **Common carps** for 2 months with curcumin, turmeric, SiO2NPs, **curcumin-loaded nanocomposites at the range of 1–40** g/kg diet	Nano-encapsulated curcumin lead to lowest metal accumulation therefore a dietary treatment with antioxidant and health protective potential	Moradi *et al.* (2023)
**(5) 32 adult male rats** were administered orally for 8 weeks with 100 mg/kg catechin or **nanocapsule niosomal form of catechin**.	Nano-encapsulated catechin yielded stronger antioxidant and antithyroid effects compared to the control (p <0.0001).	Mohamadizadeh *et al.* (2022)
**(6)** Oral administration of **male mice** were administered with quercetin-conjugated silver nanoparticles **(Q-AgNPs)**	Treatment with Q-AgNPs reduced proinflammatory and significantly increasing anti-inflammatory cytokine (IL-10).	Abdel-Hakeem *et al.* (2025)
**(7)** Nasal administration in **Wistar rats** of chitosan-coated **Rosmarinic acid nanoemulsions** (RA CNE) on lipopolysaccharide (LPS)-induced memory deficit, neuroinflammation,	RA CNE nasal treatment elicits a neuro-protective effect against LPS-induced damage and also facilitated RA bioavailability in the brain.	Silveira *et al.* (2020)
**(8)** Polymeric and lipidic nanocapsules (NCs) of **ferulic acid** which were tested in **vivo in rats**	The study revealed that ferulic acid lipid NCs can efficiently inhibit oxidative damage in rats	El-Gogary *et al.* (2022)
**(9) Dietary supplementation of rats** with ferulic acid and protein **nano-encapsulated ferulic acid**-	Ferulic acid nano formulations significantly mitigated liver toxicity	Essa *et al.* (2025)

## Nano-polyphenols

Plant-based polyphenols are well known for their various health-protection properties, which are strongly associated with their ability to efficiently scavenge free radicals (FRs), intruding the living body ( Thakur *et al.*, 2023). Among them, polyphenols, such as curcumin, resveratrol (non-flavonoids), and flavonoids, have received much attention for their ability to reduce cellular stress-induced injury. Flavonoids, in particular, are a class of phenolic compounds with well-established functional properties that are strongly related to their structure ( Kiokias & Varzakas, 2014).

Despite their wide range of biological properties, polyphenols exhibit limited stability, volatility at low pH, and poor bioavailability ( Ayala-Fuentes & Chavez-Santoscoy, 2021). A few researchers have explored the potential of nanoencapsulation as a novel technique to enhance dietary absorption of flavonoids ( Adefegha *et al.*, 2025; Bokharaeian *et al.*, 2025).

Furthermore, a body of literature has demonstrated that when flavonoids are nano-formulated, they could enlarge their applications as functional antioxidants in the manufacturing process ( Lavanya & Padmini, 2023; Xu *et al.*, 2025). Sheng *et al.* (2025) observed that sea buckthorn flavonoid-based nanoparticles achieved ~80% higher encapsulation efficiency and bio accessibility compared to control samples. An analysis of a few commonly examined nano-encapsulation-polyphenols is provided in the following paragraphs.

### Nano-flavonoids

***Nano-quercetin***

Quercetin is a flavonoid that is widespread in many natural sources such as onions, berries, apples, and kale. ( Xu *et al.*, 2019). Several studies have reviewed their antioxidant activities in various food and biological systems ( Haq & AlAmro, 2019; Liu *et al.*, 2024).

During the last decade, a body of literature has investigated novel nanotechnology-based methods to mitigate some factors that limit their pharmaceutical and technological applications, such as low solubility and bioavailability ( Attar *et al.*, 2023; Liu *et al.*, 2024).

Homayoonfal *et al.* (2024) reported that the nano-formulation of quercetin causes more remarkable anticancer effects than its free form. Furthermore, they claimed that incorporating quercetin into various nano-delivery systems improved its sustained release and stability, extended its circulation time, enhanced its accumulation at target sites, and increased its therapeutic efficiency.

In an earlier study in rat models ( Jain *et al.*, 2013), a self-emulsifying nanoformulation of quercetin exhibited a significantly higher antioxidant potential than free quercetin when evaluated as a function of the capability to combat doxorubicin- and cyclosporin A-induced cardiotoxicity and nephrotoxicity, respectively.

Silva *et al.* (2023) reported that quercetin-loaded olive oil nanoemulsions exhibit higher activity against lipid peroxidation, which is linked to skin sensitization. More recently, Wang *et al.* (2025) performed a study that yielded novel nanoparticles that enhanced the bioavailability of quercetin, thereby enabling its use in various commercial applications.

A few authors have successfully demonstrated that nanoencapsulation of quercetin has positive effects on various health applications, including: (i) stronger antibacterial properties ( Li *et al.*, 2024) (ii) mediation of brain damage caused by exposure to heavy metals ( Ghosh *et al.*, 2018) (iii) neurodegeneration induced by oxidative stress ( Nday *et al.*, 2015) (iv) stronger cytotoxicity in lymphoma cells and inhibition of doxorubicin, indicating cardiotoxicity ( Tzankova *et al.*, 2024).

Bose *et al.* (2024) examined the encapsulation of quercetin and vitamin D3 (QVD) using solid lipid nanoparticles (QVD-SLNs), which were incorporated into the scaffold to enhance bone regeneration. The QVD-SLN-loaded scaffolds showed ~4.2-fold higher in vitro chemopreventive potential against osteosarcoma cells than the control, proving their potential to treat various bone-related disorders for low- or non-load-bearing applications.

Bakr *et al.* (2024) observed that nano formulated quercetin following its administration in rats- performed better than pure quercetin in terms of anti-oxidation and anti-inflammatory properties. Similar findings on the positive impact of nanoencapsulation were drawn in another recent clinical trial in mice ( Abdel-Hakeem *et al.* (2025), which focused on neuroinflammation and pharmacokinetics. An overview of the results is given in Table 2.

***Nano-rutin***

Rutin is a plant-derived flavonoid known for its strong functional health properties and is found in buckwheat, apples, and citrus fruits. Enogieru *et al.* (2018) developed rutin-loaded caseinate nanoparticles (NPs) with high encapsulation efficacy and total antioxidant capacity.

Very recently, Lopez-Polo *et al.* (2024) showed that encapsulation of rutin in nano liposomes yielded higher encapsulation (89.3 ± 0.5%) a fact which makes it attractive for novel commercial applications in various food systems.

The data recorded from the in vitro studies showed that encapsulation into these nanosystems allowed us to overcome the photosensitivity limitation of rutin. Cristiano *et al.* (2021) confirmed the potential of rutin-loaded nanosystems in skin diseases, mainly related to their anti-inflammatory and antioxidant effects.

Sguizzato *et al.* (2025) investigated the use of nanosystems (rutin-containing cyclodextrins) for the delivery of rutin via oral administration. A dialysis study using Wistar rat small intestine fragments demonstrated an increased release of rutin from the RCL. This study revealed that rutin-containing cyclodextrin nanosystems could offer a possible strategy for dietary supplementation in diabetics.

***Nano-catechin***

Catechin is a flavonoid, specifically flavan-3-ol, known for its potent antioxidant, anti-inflammatory, and antibacterial properties, and is abundantly found in green tea, cocoa, and wine ( Wu *et al.*, 2017).

Yao *et al.* (2024) encapsulated catechin using chitosan/SDS nanoparticles, which were subsequently orally administered to rats. According to the results, nanoencapsulation yielded a statistically higher dietary absorption and bioavailability of catechin compared to placebo. Mohamadizadeh *et al.* (2022) performed a clinical trial in adult male rats and concluded that nano-encapsulated catechin increased the activity of catechin against experimental oxidative stress conditions.

### Other nano-polyphenols

***Nano-curcumin***

Curcumin is a polyphenol and the primary bioactive compound in turmeric (Curcuma longa), a spice and traditional medicine ingredient known for its bright yellow color. It is well known for its strong antioxidant properties, which are linked to the prevention and treatment of various aging-associated pathological conditions (i.e., cardiovascular and neurodegenerative diseases, type 2 diabetes, and osteoporosis) ( Sundar Dhilip Kumar *et al.*, 2018). However, its metabolic instability and hydrophobicity have hindered its clinical application, leading to short plasma half-life, poor absorption, and low bioavailability. Both in vitro and in vivo evidence suggests that nanocurcumin formulations have better health-promoting properties than conventional (native) curcumin ( Dewangan *et al.*, 2017; El-Naggar *et al.*, 2019). An interesting study by Verma *et al.* (2023) confirmed that nanocurcumin prepared through microfluidization exerted stronger antioxidant activity and stability compared to pure curcumin without a lack of safety (as demonstrated via a cytotoxicity assay).

Another study on the nanoencapsulation of curcumin ( Ye *et al.*, 2024) reported that curcumin nano-emulsions resulted in a higher retention of antioxidant activity, bioaccessibility, and absorption than pure curcumin.

Moradi *et al.*, 2023 examined whether natural zeolite nanoparticles can serve as efficient carriers of curcumin to enable its higher bioabsorption in a clinical trial in carp. This study demonstrated that nano-encapsulated curcumin can significantly enhance in vivo antioxidant effects following dietary supplementation.

***Nano-silymarin***

Silymarin is a complex of flavonoids, specifically flavonolignans, extracted from milk thistle (Silybum marianum), and is known for its potent antioxidant, anti-inflammatory, and liver-protective properties. ( Mandegary *et al.*, 2013). Parveen *et al.* (2011) reported that silymarin nano-emulsions exerted a clear inhibitory effect on lipid peroxidation following their incorporation into a rat model. Another novel study by Adhikari and Arora (2015) demonstrated the potential of nano-encapsulated silymarin to protect against radiation side effects.

***Nano-polyphenol mixtures***

Franco *et al.* (2021) encapsulated blueberry flavonoid-rich extracts using chitosan-based nanoparticles and observed stronger antioxidant effects compared to the natural preparations. Nagaich *et al.* (2016) focused on a mixture of flavonoids present in apple extracts that were nanoencapsulated by use of silver nanoparticles. According to the results, the yielded hydrogels showed 98% in vitro release whereas the flavonoid based nanoparticles exerted statistically higher antioxidant effects.

da Silva *et al.* (2025) reported that nanoparticles based on whey and soy proteins enhanced the antioxidant activity of phenolic compound extracts from cantaloupe melon pulp flour (Cucumis melo L.). Freitas *et al.* (2025) evaluated the use of the PuroSorb PAD950 resin to enrich flavonoids from Malpighia emarginata DC. pomace extract (MEPE) and its nano capsules (NC), liposomes (LP), and nanogels (NG). The authors concluded that flavonoid-based based NC (FLA-NC) had the highest encapsulation efficiency (81%), whereas FLA-LP and FLA-NG further improved antioxidant capacity compared to the non-encapsulated fraction.

The potential of nano-encapsulated polyphenol extracts to exert health benefits has been well demonstrated by the following recent studies:

- Antimicrobial effect of nanocurcumin mixed with nano-quercetin in chitosan cells ( Kunadu *et al.*, 2025);
- Combined therapeutic effect of curcumin and piperine nanoformulations against pulmonary toxicity following administration in mice ( Khederzadeh *et al.*, 2024);
- nanoencapsulation of phenolic mixtures from the peel of pomegranate fruits with clear antioxidant and antibacterial effects ( Andishmand *et al.*, 2023)

## Nano-Phenolic acids

Phenolic acids concern a range of natural compounds widespread in many aromatic plants and botanicals. which ( Kiokias & Oreopoulou, 2021). Among these, the present analysis focuses on certain phenolics (caffeic, ferulic, gallic, and rosmarinic), which have been shown to possess strong antioxidant and biochemical properties ( Kiokias *et al.*, 2020).

Felix *et al.* (2025) noted that ferulic acid (FA) and caffeic acid (CA), although well known for their biological and health protection activities, exhibit physicochemical instability that limits their broader therapeutic applications.

A similar conclusion regarding the reduced bioavailability of phenolics was drawn by Garavand *et al.*, 2021. Over the last few years, the application of nanoscience-based techniques has enabled the development of nano-carriers for the safe and efficient delivery of phenolics (nanofibers, electro-sprayed nano-particles, etc.) ( Esfanjani & Jafari, 2016). A few of the phenolic acids reviewed in the current analysis are given in Figure 2.

### Nano-rosmarinic acid (RA)

Oxidative stress and free radicals are strongly linked to the development of various neurodegenerative disorders (ND) ( Liu *et al.*, 2017). Over the last decade, several clinical studies have focused on rosmarinic acid (RA) and its potential neuroprotective properties, despite some limitations on its bioavailability ( Habtemariam, 2018).

Campos *et al.* (2014) was one of the first researchers who had perceived the idea of developing nano emulsion formulations to ameliorate the oral bioavailability of RA. Furthermore, Fachel *et al.* (2019) suggested the application of RA-loaded nanoparticles via the nasal route as a new therapeutic approach for ND. Building on the findings of earlier studies, Silveira *et al.* (2020) further optimized a novel treatment during a clinical trial in Wistar rats. The researchers reported that nasal administration of chitosan-coated RA nano emulsions exerted a clear antioxidant effect against neuro-oxidative damage, while also facilitating RA bioavailability in the brain.

The development of various types of nanoparticles as nanocarriers for the efficient delivery of RA in clinical experiments has been recently applied by various researchers in different model systems, such as:

- Chen *et al.* (2024) produced nanoparticles loaded with RA-targeting in a mouse model of radiation-induced pulmonary fibrosis.
- Imraish *et al.* (2024) prepared RA-based green metallic nanoparticles to suppress pro-inflammatory cytokines in a dose-dependent manner.
- Chhabra *et al.* (2020) developed RA-based graphene nanoparticles with higher in vitro (in vivo in rats) antibacterial activity than RA alone.
- Solak *et al.* (2025) observed significant antimicrobial properties and antioxidant activity of RA-loaded Resomer RG 502 H-based nanoparticles against tert-butyl hydroperoxide-induced oxidative stress.

Rosemary essential oil (REO) is a natural food preservative with strong antibacterial and antioxidant properties ( Amoah *et al.*, 2016). Yang *et al.* (2023) focused on various delivery systems of REO in food preservation applications, such as nano emulsions (NEs), solid particle encapsulation (SPE), and biodegradable food packaging film/coatings (BFPF/BFPC). The authors concluded that REO is being utilized for food preservation through various delivery forms, such as NEs, SPE, and BFPF, in addition to its direct application for food preservation. The ability of encapsulated REO to preserve fresh foods, such as meat, fish, fruits, and vegetables, has been shown to be significantly superior to that of conventional methods.

### Nano-ferulic acid (FA)

A few researchers have demonstrated that the nanoencapsulation of ferulic acid in nanogels ( Grimaudo *et al.*, 2020) or nanoemulsion systems ( Kuzhithariel Remanan & Zhu, 2023) can successfully increase their stability, release, and bioavailability.

Rampelotto *et al.* (2022) prepared ferulic acid (FA)-loaded nano-capsules suspensions (NC-FA) with ethyl cellulose, which showed decreased irritation, no cytotoxicity, and enhanced antioxidant activity.

Rani *et al.* (2024) investigated all trans-ferulic acid-loaded chitosan-based nanoparticles and reported positive effects in terms of radical scavenging and antibacterial properties, along with higher in vitro anti-inflammatory activity. Similarly, Abd-Allah *et al.* (2024) prepared ferulic acid (FA) nanoemulsions and demonstrated their efficacy in achieving a sustained release of ferulic acid in a gastric model system, as well as stronger anti-inflammatory and in vivo antioxidant properties.

Furthermore, the following studies have also concluded the beneficial health effects of FA nano-encapsulation in the development of novel treatments:

- Ferulic acid (FA)-loaded FU nanoparticles have the potential to protect against kidney damage by suppressing cisplatin-induced DNA damage ( Gao *et al.*, 2022);
- Polymeric and lipidic nanocapsules (NCs) of ferulic acid showed (i) clear in vitro anticancer activity when tested in colorectal cancer cell lines and (ii) strong in vivo anti-inflammatory action following a clinical trial in rats ( El-Gogary *et al.*, 2022).
- nano-encapsulated ferulic acid shows a clear inhibitory effect against in vivo oxidative DNA damage in rats and thereby protects against liver toxicity ( Essa *et al.*, 2025).

**Figure 3.**
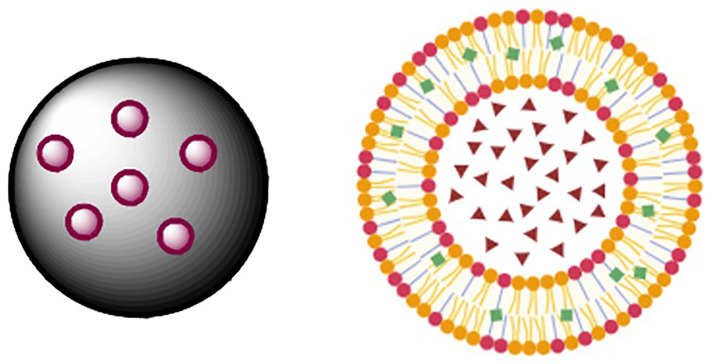
Polymeric and lipidic nano capsules of ferulic acid. *(kindly provided by Prof. Efthymiadi, National Kapodistrian University of Athens)*.

The Figure 3 presents images of the polymeric and lipid nano capsules of ferulic acid.

### Nano-caffeic acid (CA) and esters

Tiwari *et al.* (2023) designed a graphene oxide (CA)-based nanocomposite that achieved an efficient release of caffeic acid. Salehi *et al.* (2025) conducted an in vitro study and concluded that nano-caffeic acid particles have stronger antifungal efficacy against candidiasis (a widespread fungal infection) compared to natural caffeic acid.

During the last few years, researchers have focused on caffeic acid phenethyl ester (CAPE), which has also been reported to possess beneficial health properties. Guan *et al.* (2019) nano-encapsulated CAPE in aqueous propylene glycol (PG) using a temperature cycle method. Nanoencapsulation enhanced the cytotoxicity of CAPE against colon cancer HCT-116 and breast cancer MCF-7 cells, and thymol additionally enhanced the cytotoxicity of CAPE dispersions.

Colpan & Erdemir (2021) synthesized quercetin-caffeic-acid phenethyl ester (CAPE)-co-loaded poly(lactic-co-glycolic-acid) (PLGA) nanoparticles (QuCaNPs) and investigated their anticancer activity. The authors concluded that QuCaNPs exhibited improved antitumor activity in human colorectal carcinoma HT-29 cells.

### Other nano-phenolic acids

In a recent study, gallic acid (GA)-loaded nanocomposites with high water solubility were synthesized by Feng *et al.* (2024) via solvent evaporation using food-grade silica (F-SiO2) as the carrier. According to researchers, encapsulation of GA effectively prevented the self-aggregation phenomenon, thereby facilitating the exposure of its active phenolic hydroxyl group and significantly enhancing its water-based biological activity. Dorniani *et al.* (2016) formulated graphene oxide (GO) nanoparticles to encapsulate gallic acid (GA) and reported a clear inhibitory effect on the growth of cancer cells.

Okumus *et al.* (2024) observed that maltodextrin-based nanoencapsulation of ellagic acid enhances the bioavailability of this phenolic compound, opening a pathway for novel food and nutraceutical applications.

Kamal *et al.* (2021) developed a ρ-coumaric acid-chitosan-based nanogel loaded with Syzygium aromaticum essential oil (SAEO). The authors reported that nanoencapsulation resulted in much stronger antioxidant and chemotherapeutic activities than those of the essential oil.

More recently, Manasa *et al.* (2024) developed nanoformulations of vanillic acid that exhibited a perfect encapsulation efficiency of 93% and smooth microstructural characteristics as shown by Scanning Electron Microscopy.

## Conclusions and challenges

This analysis reviewed the most updated research data (mainly over the last five years) concerning the health protective properties of various nano-formulated natural antioxidants. In summary, the following conclusions were drawn:

-Available literature evidence supports that the application of various nano-based systems can improve the solubility, stability, and biological properties of natural bioactive antioxidants (including tocopherols, carotenes, xanthophylls, polyphenols, and phenolic acids).

-A range of plant based nano-formulations of natural antioxidants were shown to exert stronger antioxidant properties than formulations fabricated by traditional extracted natural compounds.

-Plant extract-based nanoparticles have been reported to exert strong biological activities, including antifungal and anticancer effects, with improved pharmacokinetics, tissue targeting, and reduced side effects. In particular for the phenolic acid extracted from natural Rosemary preparations (Rosmarinic acid-RA), recent clinical evidence has revealed its potential for the treatment of Neurodegenerative disorders (ND).

However, an important challenge still occurs concerning the safety aspects of nanoencapsulation that must be carefully assessed to exclude potential adverse health effects. Therefore:

- Further research is needed to ensure that nanodelivered phytonutrients are non-toxic, organ/tissue specific, on time in situ release, easy to use, and have a high radical scavenging function.
- Commercialization of food products containing nanocarriers with nutraceuticals is still in the primary stages of expansion. Specific trials on pharmacokinetic parameters and biological processes should be undertaken to design appropriate and safe nano-antioxidants.

## Informed consent statement

The views expressed in this publication are purely those of the writer and may not be regarded as stating the official position of the European Commission.
