# Supplementary material for: Antioxidant properties of nano-formulated carotenoids and natural phenolic compounds against oxidative stress-induced health diseases
Source: Open Res Eur. 2026 Feb 24;6:11. Originally published 2026 Jan 6. [Version 2] doi: 10.12688/openreseurope.21957.2 (PMC12982999; doi:10.12688/openreseurope.21957.2)
Supplement: Peer review history

# Reviewer report: Reviewer response for version 2

**DOI:** 10.21956/openreseurope.24954.r70683

**Published:** 2026-03-12

**Authors:** Neihaya Heikmat Zaki

**Affiliations:** 1 Biology, Mustansiriyah university, college of science, Baghdad, Baghdad, Iraq

-Abbreviations should be written completely the first time.

 -No abbreviation should be written in the beginning of the paragraph.

 -Mechanisms of these nano compounds as antioxidants must be mentioned in the review.

 -Mention the cytotoxicity of these nano compounds in vitro and in vivo.

 -Mention the diseases that are induced by oxidative stress.

Is the review written in accessible language?

Yes

Are all factual statements correct and adequately supported by citations?

Yes

Are the conclusions drawn appropriate in the context of the current research literature?

Yes

Is the topic of the review discussed comprehensively in the context of the current literature?

Yes

Reviewer Expertise:

Biotechnology, nanotechnology, microbiology, enzymology.

I confirm that I have read this submission and believe that I have an appropriate level of expertise to confirm that it is of an acceptable scientific standard.

# Reviewer report: Reviewer response for version 2

**DOI:** 10.21956/openreseurope.24954.r70685

**Published:** 2026-03-10

**Authors:** Shobana Chandrasekar

**Affiliations:** 1 Vels Institute of Science, Technology and Advanced Studies (VISTAS), Chennai, Tamil Nadu, India

The manuscript presents a comprehensive review of the antioxidant potential of nano-formulated carotenoids and natural phenolic compounds in combating oxidative stress-related diseases. The topic is timely and relevant, as oxidative stress plays a significant role in the development and progression of many chronic disorders, including cardiovascular diseases, diabetes, neurodegenerative conditions, and cancer. By integrating concepts from nanotechnology and natural product research, the article highlights emerging strategies to improve the bioavailability, stability, and therapeutic efficiency of natural antioxidants.

 The manuscript successfully summarizes current knowledge regarding carotenoids and phenolic compounds and discusses different nano-delivery systems such as liposomes, nanoemulsions, and polymeric nanoparticles. The review provides useful insights into how nano-formulation can enhance the biological activity and clinical potential of these bioactive compounds. The information presented is relevant for researchers working in the fields of nanomedicine, pharmacology, nutrition, and biomedical sciences.

 Overall, the article contributes valuable information to the growing body of literature on nanotechnology-based antioxidant therapies. The manuscript is well aligned with current research trends and offers a useful reference for future studies in this area. Therefore, the manuscript can be considered suitable for indexing.

Is the review written in accessible language?

Yes

Are all factual statements correct and adequately supported by citations?

Yes

Are the conclusions drawn appropriate in the context of the current research literature?

Yes

Is the topic of the review discussed comprehensively in the context of the current literature?

Yes

Reviewer Expertise:

Clinical Nutrition, Neuroscience

I confirm that I have read this submission and believe that I have an appropriate level of expertise to confirm that it is of an acceptable scientific standard.

## References

[1] : Therapeutic Potential of Flavonoids in Diabetes Mellitus Management: Molecular Insights and the Future Directions for Drug Design. *Current Drug Discovery Technologies* .2026;23(1) : 10.2174/0115701638333208250522050837 10.2174/0115701638333208250522050837 40457969

# Reviewer report: Reviewer response for version 1

**DOI:** 10.21956/openreseurope.23754.r68241

**Published:** 2026-01-20

**Authors:** Emine Okumuş

**Affiliations:** 1 Van Yuzuncu Yil University, Van, Turkey

The abstract should be improved in terms of the importance of the topic.

 Abbreviations should be explained with their full name the first time they appear, and then used in their abbreviated form (e.g., ROS).

 ‘Several recent studies have explored the effect of nanoencapsulation on the bio-accessibility of polyphenols from botanical extracts ( Ferreira Dantas *et al.*, 2025; Kato *et al.*, 2023; Myint *et al.*, 2021)’.

 This sentence should be elaborated upon with study results detailing its effects.

 For example,

 Okumus, E. Green synthesis of silver nanoparticles using *Hebeloma excedens* mushroom extract as a new source: Anti-lipid peroxidation, bioaccessibility and antidiabetic properties. *Food Measure* **18**, 5157–5169 (2024). https://doi.org/10.1007/s11694-024-02635-2

 Okumus, E., Temiz, M.A. Use of hazelnut by-products as a sustainable source for nanoparticle synthesis: characterization, bioactive components and health effects of maltodextrin-coated nanocapsules. *Food Measure* (2025). https://doi.org/10.1007/s11694-025-03912-4

 Important note: The above references are shared with the author only as examples and are advisory in explaining the mechanism underlying the hypothesis presented in the manuscript. The use of these references is not mandatory.

 Specifying the application doses in clinical studies will make it easier for the reader to make comparisons (e.g., Olim *et al.* (2021) performed a very interesting biochemical study concluding that tocopherol-based cholesterol doxorubicin prodrug conjugates could offer promising nano formulations for use in breast cancer chemotherapy.)

 It is recommended to add information regarding dosage and toxicity to the ‘Conclusions and challenges’ section.

Is the review written in accessible language?

Yes

Are all factual statements correct and adequately supported by citations?

Yes

Are the conclusions drawn appropriate in the context of the current research literature?

Yes

Is the topic of the review discussed comprehensively in the context of the current literature?

Yes

Reviewer Expertise:

Nanoparticle synthesis, Green synthesis, Phytotherapy, phenolics and antioxidants.

I confirm that I have read this submission and believe that I have an appropriate level of expertise to confirm that it is of an acceptable scientific standard, however I have significant reservations, as outlined above.
